# Accurate Correction of the “Bulk Response” in Surface Plasmon Resonance Sensing Provides New Insights on Interactions Involving Lysozyme and Poly(ethylene glycol)

**DOI:** 10.1021/acssensors.2c00273

**Published:** 2022-03-17

**Authors:** Justas Svirelis, John Andersson, Anna Stradner, Andreas Dahlin

**Affiliations:** † Department of Chemistry and Chemical Engineering, Chalmers University of Technology, SE-41296 Gothenburg, Sweden; ‡ Division of Physical Chemistry, Lund University, SE-22100 Lund, Sweden

**Keywords:** surface plasmon resonance, poly(ethylene glycol), lysozyme, kinetics, quantification

## Abstract

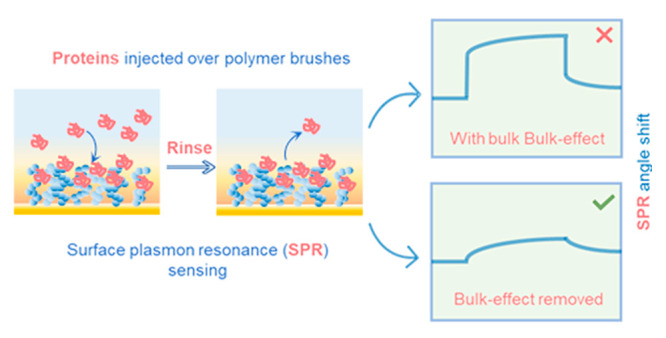

Surface plasmon resonance is a very well-established surface sensitive technique for label-free analysis of biomolecular interactions, generating thousands of publications each year. An inconvenient effect that complicates interpretation of SPR results is the “bulk response” from molecules in solution, which generate signals without really binding to the surface. Here we present a physical model for determining the bulk response contribution and verify its accuracy. Our method does not require a reference channel or a separate surface region. We show that proper subtraction of the bulk response reveals an interaction between poly(ethylene glycol) brushes and the protein lysozyme at physiological conditions. Importantly, we also show that the bulk response correction method implemented in commercial instruments is not generally accurate. Using our method, the equilibrium affinity between polymer and protein is determined to be *K*_D_ = 200 μM. One reason for the weak affinity is that the interaction is relatively short-lived (1/*k*_off_ < 30 s). Furthermore, we show that the bulk response correction also reveals the dynamics of self-interactions between lysozyme molecules on surfaces. Besides providing new insights on important biomolecular interactions, our method can be widely applied to improve the accuracy of SPR data generated by instruments worldwide.

Surface plasmon resonance (SPR) is the most established method for label-free biomolecular interaction analysis. SPR makes it possible to determine the affinity of an interaction and to probe binding kinetics in real-time, with one molecule on the surface and the other introduced in solution. The widespread impact of SPR in molecular biology and elsewhere is evident.^1−3^ The high resolution in surface coverage (sometimes below 0.1 ng/cm^2^) also makes SPR sensors interesting for detection of analytes from complex samples, although it is highly challenging to immobilize receptors with sufficient affinity while also preventing nonspecific binding of other molecules in the sample.^4^

An inconvenient issue with SPR sensing is that although the method is “surface sensitive”, the evanescent field extends hundreds of nanometers from the surface, i.e., much more than the thickness of the typical analytes (e.g., proteins ranging from 2 to 10 nm). This means that when molecules are injected, even those that do not bind to the surface will give a response, especially when high concentrations are introduced, which is necessary for probing weak interactions. Similarly, if a complex sample is injected, the bulk liquid refractive index (RI) will change considerably, and a large but false sensor signal is observed. This “bulk response” problem, which often also applies to other plasmonic sensor geometries than planar films,^5^ has haunted SPR users for decades, as it is difficult to separate from the real signal, i.e., that originating from surface binding. Arguably, the bulk response effect is one major reason many of the SPR publications generated every year (thousands) actually have questionable conclusions.^6^ To address this problem, users have sometimes introduced a separate reference channel to measure the bulk response.^7,8^ However, this requires that the reference channel surface perfectly repels the injected molecules (no adsorption), and even then an error will be introduced unless its coating has identical thickness to that in the sample channel.^9^ Naturally, it is preferable to obtain the bulk contribution to the SPR signal from the very same sensor surface in order to eliminate such variations compared to the reference. To the best of our knowledge, the only previous work that systematically aimed to remove the bulk response is studies by Chinowsky and Yee et al.^10,11^ Their work correctly pointed out the importance of utilizing the total internal reflection (TIR) angle to obtain the bulk RI. However, the method still used a separate surface region to obtain the TIR angle. To date, focus has been on improving instrument stability by compensating for spontaneous fluctuations in the bulk RI, such as temperature variations,^12^ rather than accounting for the molecules in the bulk.

Notably, commercial instruments have very recently implemented features for removing the bulk response upon injections (see PureKinetics by Bionavis), which calls for investigations of their validity and applicability. We are only aware of one study that explicitly mentions that the built-in method of a commercial instrument is utilized: Kari et al., who studied corona formation on immobilized vesicles.^13^ However, without questioning their conclusions, we note that their data clearly showed remaining bulk responses during injections. There appears to be no systematic investigation that provides a simple model of the bulk contribution to the SPR response and clarifies how it can be removed, thereby proving new insights into weak molecular interactions.

In this work, we present a new method for direct bulk response correction in SPR, without the requirement of any reference region/channel. We implement the method in order to analyze the low affinity interaction between the protein lysozyme (LYZ) and grafted poly(ethylene glycol) (PEG). This interaction is of broad interest for several reasons: First, since PEG is generally protein repelling, the interaction is quite special and may even be considered controversial. Second, the interaction has medical relevance, since LYZ is abundant in some bodily fluids (saliva, tears, etc.) and PEG is a common component in biomedical devices. Indeed, it has long since attracted interest from theoreticians.^14^ We show that the PEG-LYZ interaction is an excellent model system for illustrating the importance of bulk response correction in SPR. In particular, we illustrate that the thickness of the layer with receptors existing on the surface must be taken into account. After fully characterizing the system, the bulk response is accounted for by a simple analytical model that uses the TIR angle response as the only input. The corrected data gives the equilibrium affinity and kinetics of the PEG-LYZ interaction as well as LYZ self-interactions.

## Experimental Section

### Chemicals

Thiol-terminated PEG with an average molecular weight of 20 kg/mol (PDI < 1.07) was purchased from LaysanBio. Lysozyme (LYZ) from chicken egg white (purity ≥90%; L6876) and bovine serum albumin (BSA, purity ≥96%) from Sigma-Aldrich were used without further purification. HCl and phosphate buffer saline (PBS) tablets (137 mM NaCl, 10 mM Na_2_HPO_4_, 2.7 mM KCl) were purchased from Sigma-Aldrich. The PBS buffer was degassed and filtered (0.2 μm hydrophilic nonsterile Sartorius Minisart filters) before use. Hydrogen peroxide (H_2_O_2_, 30%) was purchased from Merck and ammonium hydroxide (NH_4_OH, 28–30%) from Thermo Fischer Scientific. EtOH was from Solveco. The water used in this study was ASTM Research grade Type I ultrafiltered water (18.6 MΩ). Note that the data presented in this work is obtained from a single source of LYZ: product no. L6876, lot # SLBZ8428. Experiments on other LYZ batches indicated similar results (not shown). To investigate any potential effects from the preparation of the LYZ solutions, we also tested dissolving the protein in pure water before exposing it to salt (as opposed to direct dissolution in PBS) and to filter the solution through a 0.2 μm low protein binding syringe filter before the injections in SPR. This did not have any noticeable effect on the SPR signals. To verify the concentrations, the absorbance (log_10_) of a solution prepared at 1 g/L was measured to ∼2.3 at 280 nm. Using an extinction coefficient of 2.64 L/g^15^ (1 cm path length), this gives an actual concentration that is only 10% lower. All concentrations stated are those based on measured weight and volume.

### Sensor Chip Preparation

SPR chips with ∼2 nm Cr and 50 nm Au (the optimal thickness for a narrow and deep SPR minimum) were prepared by electron beam heated physical vapor deposition (Lesker PVD 225) on glass substrates (Bionavis) cleaned using RCA2 (1:1:5 volume of conc. HCl:H_2_O_2_ (30%):H_2_O at 80 °C) and 50 W O_2_ plasma at 250 mTorr. Prior to experiments, the surfaces were cleaned with RCA1 cleaning solution, containing 5:1:1 v/v ratio MQ water, H_2_O_2_, and NH_4_OH, accordingly, at 75 °C for 20 min, followed by 10 min incubation in 99.8% EtOH and blowing with N_2_.

### PEG Grafting

Twenty kg/mol thiol-terminated PEG was grafted on planar gold SPR sensors at 0.12 g/L concentration in freshly prepared and filtered 0.9 M Na_2_SO_4_ solution for 2 h under 50 rpm stirring. When the grafting finished, the sensors were thoroughly rinsed with MQ water and dried with N_2_. Functionalized SPR sensors were left immersed in MQ water on a Teflon stand overnight.

### SPR Experiments

Experiments were conducted with an SPR Navi 220A (BioNavis) instrument with the temperature set to 25 °C. The single poly(ether ether ketone) flow cell has two flow channels operated in parallel mode and multiple wavelengths. All data presented was obtained at 670 nm. Scans in air to determine dry PEG thickness were obtained before measuring in liquid. All protein injections were done in ordinary PBS buffer at a flow rate of 20 μL/min.

### Data Analysis

The dry thickness and exclusion height of the PEG brushes was determined by Fresnel model fits to the SPR spectra as described previously.^16^ In brief, the thicknesses and refractive indexes of metal layers were determined independently using SPR chips without the PEG film. Once the parameters were confirmed and gave a reasonable fit, the same values were used when fitting the spectra after introducing an additional layer representing the PEG. Similarly, to determine the height of the hydrated PEG brush, the Fresnel models were used in the absence and presence of BSA (a non-interacting protein) in the liquid bulk.^17^ For equilibrium analysis, all LYZ concentrations except the lowest were measured repeatedly (Table S1). A linear baseline correction was performed if the drift was the same throughout the experiment (typically <10^–4^ °/min). For the lowest concentrations (<0.1 g/L), the error bars were set to twice the noise level in the instrument, which is ∼0.001° for the SPR angle. Each SPR signal was corrected with its corresponding TIR angle signal and the calculation of average and standard deviation was performed afterward, for each LYZ concentration. A very small shift of ∼0.002° was normally seen for both the SPR and TIR angles when performing injections even when the protein concentration approached zero. (This can be attributed to artifacts from the liquid injection itself, such as a small temperature change.) To compensate for this (very minor) effect, this value was subtracted from all SPR and TIR angle signals in the equilibrium analysis.

## Theory and Background

**Figure 1:**
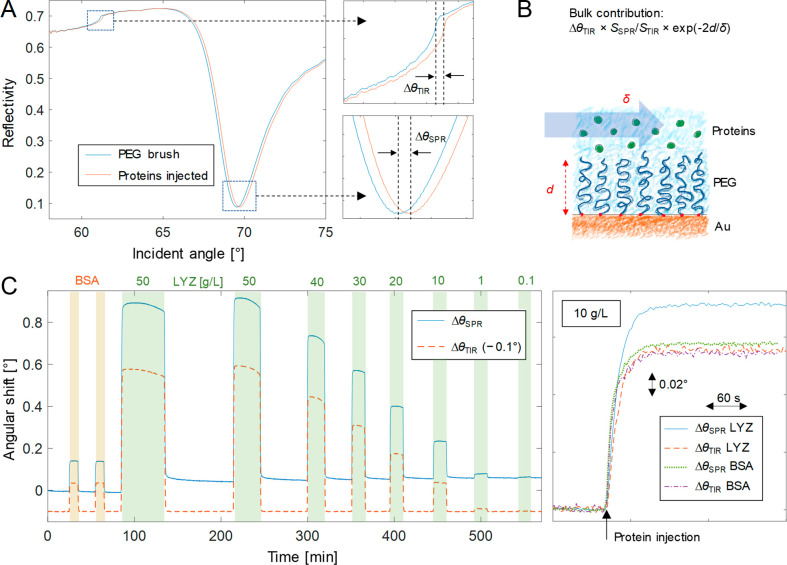
(A) Angular SPR spectrum and its changes upon injection of a high concentration of proteins to a PEG brush, causing shifts in the TIR and SPR angles. (B) Conceptual illustration of the bulk response generated by the proteins in the liquid bulk solution above the PEG brush. The thickness *d* represents the region from which the bulk molecules are excluded, and δ is the decay length of the evanescent field. (C) Typical SPR sensorgram when injecting BSA (10 g/L) and LYZ at various concentrations. The TIR angle trace is also shown (offset). To the right is shown in further detail the SPR and TIR angle signals for one BSA and one LYZ injection (both at 10 g/L). (The irreversible binding of LYZ was already maximized from previous injections.) Note that for BSA, the SPR and TIR signals are similar, while for LYZ the SPR signal is higher.

We begin by describing the theory that forms the basis for our method for bulk response correction. For well-hydrated films (such as the PEG brushes in this work), an effective field decay length can be used to quantify the SPR response.^18^ The generic expression for the SPR signal (resonance angle shift) due to a RI change can then be written as
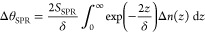
1Here, *S*_SPR_ is the bulk sensitivity (deg per RI unit), and δ is the distance from the metal where the evanescent field has decreased by a factor exp(−1). In principle, eq 1 is a linearization; i.e., the changes in RI must not be too large. However, in practice it normally holds true as long as the bulk solvent is the same (e.g., only aqueous solutions). Now, assume that a molecule binds in a region from *z* = 0 to *z* = *d* and that there is a considerable “bulk effect” from molecules at *z* > *d*. The integral can then be split into two terms:
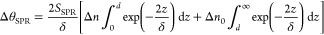
2Here, Δ*n* is the RI change inside the film of thickness *d*, and Δ*n*_0_ is the RI change in the bulk liquid due to the injected molecules. The bulk response typically becomes significant at ∼100 μg/mL or higher injected concentrations. Utilizing that the TIR angle in the spectrum (Figure 1A) is a direct reporter of *n*_0_, we have
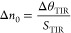
3Here, *S*_TIR_ is the sensitivity for the TIR angle. Inserting this and evaluating the integrals gives
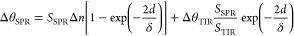
4

The two terms in eq 4 represent surface binding and the bulk response, respectively. Note that Δθ_TIR_ = 0 leaves only the term corresponding to molecules bound to the surface. Hence, the corrected SPR signal is given by
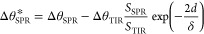
5

The bulk response correction is conceptually illustrated in Figure 1B. Importantly, in the limit of *d* ≪ δ the factor exp(−2*d*/δ) ≈ 1, which means that the SPR signal can be corrected simply by subtracting the TIR angle signal multiplied by *S*_SPR_/*S*_TIR_. This appears to be the method implemented in the commercial multiparameter SPR instrument used in this work. (The software does not describe the method, but the values generated are in agreement with this model.) At 670 nm where we perform the analysis, we determined *S*_SPR_ = 116° per RI unit and *S*_TIR_ = 74° per RI unit experimentally (Figure S1). Furthermore, we obtained δ = 226 nm by calculating the surface plasmon dispersion relation and field distribution in water^19^ (Figure S2). The values of *S*_SPR_ and δ are very similar (a few precent difference) to those in previous work,^20^ and our value of *S*_TIR_ is comparable to that obtained from linearizing Snell’s law in water with *n*_0_ = 1.33 and 1.52 for the glass prism (80° per RI unit). Finally, we used *d* = 40 nm (39.9 ± 3.8 from 11 samples) as the height of the zone that the unbound bulk molecules are excluded from. This “exclusion height” of the PEG brush is in agreement with our previous work and calculated by Fresnel models.^16,17^

Turning to the molecular interaction that we investigate, early work observed increased the affinity of lysozymes with PEG-modified surfaces and nanostructures in comparison with other proteins.^21,22^ Some later studies have explicitly suggested in a qualitative manner that LYZ interacts with PEG brushes, for instance, by hydrogen bonds.^23−26^ A few papers have reported equilibrium affinity constants: Furness et al. obtained the extremely high dissociation constant *K*_D_ = 76 mM at pH 4.0 by NMR and proposed hydrophobic interactions as the main driving force.^27^ Again by NMR, Wu et al. measured considerably higher affinities (*K*_D_ in μM region), but also found comparable or even higher binding of the BSA protein to PEG.^28^ A followup study showed that the PEG-LYZ affinity depends on the molecular weight of the PEG.^29^ To the best of our knowledge, no attempt to analyze the kinetics of the interaction has been performed. Overall, we believe previous literature clearly illustrates how difficult it is to detect and quantify interactions that are fast and/or weak.

## Results and Discussion

To study the LYZ-PEG interaction by SPR, we prepared PEG brushes on plain gold sensors by grafting thiol-terminated 20 kg/mol PEG at 0.9 M Na_2_SO_4_, as described previously.^30^ The resulting brushes had a dry thickness of 8.0 ± 1.2 nm and a grafting density of 0.25 ± 0.04 nm^–2^ determined by SPR spectra in the dry state, as in previous work.^16,31,32^Figure 1C shows the typical response in terms of SPR and TIR angles during repeated injections of LYZ to the PEG brushes in ordinary PBS buffer (pH 7.4 and physiological salt). Two things can be concluded immediately: First, the TIR angle responses are comparable to the SPR signals, which is a clear indication that a major part of the SPR response likely originates from LYZ molecules in the bulk liquid. Second, some irreversible binding occurs over the course of many injections, approaching a saturation at Δθ_SPR_ ≈ 0.1°. We attribute this to LYZ molecules that reach the underlying gold surface and adsorb irreversibly in between the PEG chains, similarly to what we recently described for polymer–polymer interactions.^16^ The signal from irreversible binding after sufficient exposure to LYZ was slightly lower than that from a monolayer formed directly on gold (Figure S3), in agreement with PEG partly preventing primary adsorption to gold. Indeed, the protein is only ∼2 nm in size^23^ and should be able to fit in between grafting sites. Tentatively, the reason LYZ reaches the underlying gold is *not* because it is a relatively small protein, but because of the interaction with PEG. In this view, the brush is a kinetic barrier for the proteins^31^ which can be overcome by favorable interactions.^33^ This view is supported by a control experiment with ubiquitin, an even smaller protein (8.5 kg/mol), which did *not* exhibit the same effect (Figure S4).

In order to further verify the interaction between LYZ and PEG, we measured the signals in SPR and TIR angles and compared them to the responses when injecting BSA, which is not interacting with the brushes.^9,30−32^ The plots to the right in Figure 1C show Δθ_SPR_ and Δθ_TIR_ for LYZ and BSA injected at the same concentration. In the case of BSA, the TIR angle response is almost identical with the SPR angle response. This is because the PEG brush excludes the protein from a certain volume^9,30^ (thickness *d*), which compensates for the differences in *S*_SPR_ and *S*_TIR_ (see Figure S1). In contrast, when LYZ is injected, the saturated signal is considerably higher in θ_SPR_ than in θ_TIR_, which can only be explained by binding. (Strictly, binding would then mean LYZ molecules appearing somewhere in the region *z* < *d*.) The TIR angle signals are similar, since all proteins have similar molar refractometry.^34^ We determined 0.173 g/cm^3^ for LYZ at pH 7.4 (Figure S5), which is similar to previous reports.^23^ Although we could confirm an interaction, it is also evident that the bulk response dominates the signal in θ_SPR_. Thus, the LYZ-PEG model system is clearly challenging to analyze accurately, especially when it comes to the affinity of the reversible binding.

We determined the equilibrium affinity between LYZ and the PEG brushes by assuming a simple Langmuir model, utilizing that the surface coverage Γ is proportional to the SPR response from surface binding. The equilibrium condition is given by
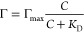
6Here Γ_max_ is the maximum surface coverage of LYZ, i.e., a saturated PEG brush. To avoid contributions from the irreversible LYZ binding (primary adsorption), we consistently extracted signals in θ_SPR_ after this response had saturated and/or from the dissociation phase. (This response is per definition the reversible part.) We then investigated three different ways to take into account the bulk response: no correction, correction according to the built-in method of the instrument, and finally correction according to eq 5, i.e., by subtracting Δθ_TIR_ × *S*_SPR_/*S*_TIR_ × exp(−2*d*/δ). We emphasize that for this method, all necessary parameters (*S*_SPR_, *S*_TIR_, *d*, and δ) were determined independently and their values implemented without alteration.

**Figure 2:**
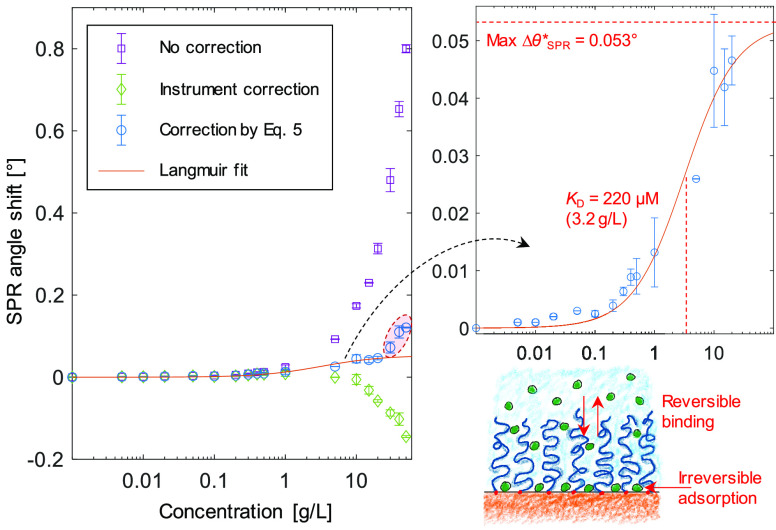
Equilibrium analysis. The signals were extracted from multiple experiments where LYZ was injected at different concentrations. The signals correspond to the reversible protein interaction with the PEG brush (not the irreversible adsorption to gold). The effects of using different methods for bulk response correction are shown. The plot to the right shows a fit to a Langmuir isotherm, using the accurate method and excluding the three highest concentrations. Error bars represent two standard deviations.

As shown in Figure 2, it is only our method of bulk response correction that gives equilibrium signals in reasonable agreement with a Langmuir isotherm. If no bulk response correction is performed, the signals keep increasing to unreasonably high values, as expected. However, when using the built-in correction method, which subtracts Δθ_TIR_ × *S*_SPR_/*S*_TIR_ (no exponential factor), the signals are even less reasonable as they become negative. This shows that the bulk response correction must be performed in a manner that accounts for the thickness of the layer present on the surface. The “commercialized” method can only be accurate for a very thin receptor layer on the metal surface or for direct protein adsorption. This is rarely the case in SPR experiments if one considers, for instance, advanced brushes for detection in complex samples^4^ or the standard dextran matrix coating.^35^ Furthermore, it was not possible to find any correction factor that (when multiplied with Δθ_TIR_) gave a flat line in the plot of equilibrium signals vs LYZ concentration, which again confirms that the interaction is real.

We observed some deviation from the Langmuir model for the very highest LYZ concentrations >20 g/L (marked data points in Figure 2), even after correcting the SPR response. These concentrations correspond to volume fractions of 2–4%, which should be low enough for osmotic effects on the brush to be negligible. Instead, we attribute the deviations from the simple Langmuir equilibrium curve to the onset of transient protein cluster formation (see also Figure 4 below). Such so-called equilibrium clusters have been observed experimentally for LYZ at higher concentrations.^36,37^ At lower volume fractions corresponding to the concentration range where we observe the deviations from the Langmuir model, simulations predicted primarily the formation of transient dimers and trimers coexisting with monomers.^36^ It should be noted, however, that previous studies have not investigated LYZ at exactly the same conditions in terms of pH and ionic strength (despite the fact that we use ordinary PBS buffer). Regardless, formation of protein clusters is consistent with the TIR angle time trace, which always decreased slowly during injections at the three highest LYZ concentrations (Figure 1), suggesting processes in the bulk solution. When some of the proteins are no longer present as monomers, the affinity to PEG is expected to be higher, and the Langmuir model is not applicable to multiple binding species. Therefore, when fitting the model (eq 6) to the data, concentrations above 20 g/L were excluded. The resulting fit (*R*^2^ > 0.98) gave *K*_D_ = 220 μM (molecular weight 14 400 g/mol for LYZ) and a saturated change in θ*_SPR_ of 0.053°. Given the purity of the LYZ, the actual *K*_D_ may be up to 10% lower, i.e., *K*_D_ ≈ 200 μM.

The relatively low saturation signal of 0.053° is likely because LYZ binds at quite some distance from the surface (∼40 nm), in the thinner outer regions of the PEG brush. In order to insert a protein into the brush, the favorable interactions with the monomers must overcome the entropic penalty of insertion.^38^ Due to the parabolic density profile of the brush, the free energy cost becomes higher for deeper insertion. Previous work with neutron reflectometry and antibodies that bind to PEG has illustrated this effect.^39^ Naturally, this also means that the *K*_D_ most likely depends on the properties of the PEG brush, and strictly speaking, a PEG brush is more like an ensemble of binding sites with different affinities at different *z* (i.e., depending on monomer density). Such effects could be investigated by varying parameters such as molecular weight and grafting density,^26^ although that is beyond the scope of the current work. Here the main purpose is to describe the method for bulk response correction and illustrate its importance for accurate quantitative analysis, regardless of what model is used to describe the interaction.

**Figure 3:**
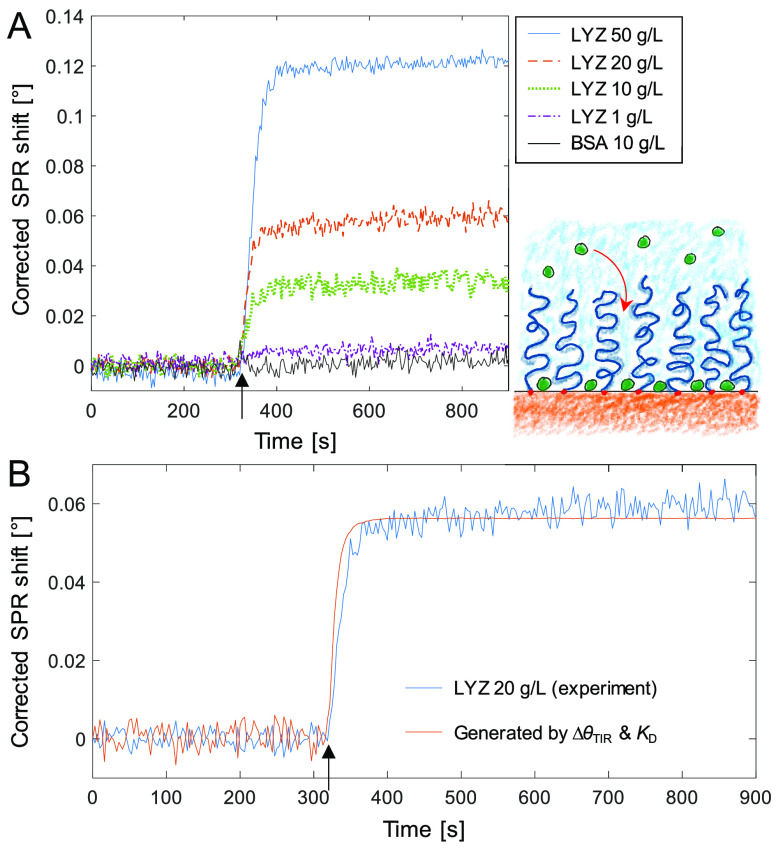
Kinetic analysis. (A) Accurate binding kinetics for LYZ at different concentrations obtained by subtracting the bulk response. (B) Example of pseudo kinetics (equilibrium at every point in time) due to the dynamics of liquid exchange in the system. The bulk response corrected SPR angle trace for 20 g/L LYZ can be recreated from the TIR angle time trace and the dissociation constant. Arrows indicate when proteins start flowing over the surface.

Turning to the kinetics of the LYZ-PEG interaction, Figure 3 shows an example of bulk response corrected (according to eq 5) sensorgrams during protein injections. It is clear that Δθ*_SPR_ is much lower than Δθ_SPR_ (compare with Figure 1). Additionally, the injections of non-interacting BSA became almost fully unnoticeable in the time trace of Δθ*_SPR_, which verifies the high accuracy of the method. Note that regardless of the construct on the surface, this kind of control is key to confirming that the correction works as it should: injecting a non-interacting molecule should give a signal in θ_SPR_ but not in θ*_SPR_.

After removing the bulk response it is possible to analyze the kinetics to find which model best describes the binding rate. The most common way is to assume first-order reaction kinetics by the differential Langmuir model:
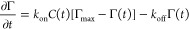
7

Note that *K*_D_ = *k*_off_/*k*_on_. Furthermore, *C*(*t*) is the concentration of species in solution at the surface, which may be influenced by mass transport.^40^ If there is no gradient in *C* in the direction perpendicular to the surface (no delay from diffusion), *C* will be linearly related to the TIR angle response:
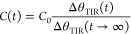
8Here *C*_0_ is the concentration in the solution which is being injected. Using eq 8 one can, in principle, solve the differential eq 7 numerically by evaluating finite time steps forward, using the TIR angle values as input to obtain *C*(*t*). However, if the LYZ-PEG equilibrium is established much faster than the concentration is altered in the liquid bulk, the system will exhibit pseudo kinetics according to the condition in eq 6:
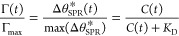
9

We found that eqs 8 and 9 were sufficient to describe the kinetics of the interaction; i.e., the time-dependence of the SPR response can be accurately modeled by taking the TIR angle into account in two ways: first, by removing the bulk contribution and then by obtaining a value for *C*(*t*). An example of a fit to Δθ*_SPR_ for 20 g/L LYZ is shown in Figure 3B. Analyzing the dissociation phase gave the same result. In other words, the rate constants *k*_on_ and *k*_off_ are simply too high for the association/dissociation to cause a significant delay in the binding/unbinding process. Therefore, Γ(*t*) is dictated by the liquid exchange system, and the bulk concentration varies primarily in time and not *z*. (There is likely a gradient in *C along* the flow channel, but not perpendicular to the surface.) This illustrates an important pitfall for kinetic analysis as it is tempting to directly fit Langmuir kinetics to the data trace of Δθ*_SPR_ (or worse, Δθ_SPR_). This leads to an excellent fit which provides no useful information whatsoever about the interaction. Instead, the only valid conclusion is that [*k*_on_*C* + *k*_off_]^−1^ and [*k*_off_]^−1^ are both lower than the characteristic time of liquid exchange, which is ∼30 s in our system (at the relatively low flow rate used). This means that *k*_off_ > 0.033 s^–1^, and hence we can conclude that the interaction is quite short-lived. Using our value of *K*_D_, we can also calculate that *k*_on_ > 150 M^–1^ s^–1^, but this does not provide so much information as a lower limit value because association rate constants are typically higher.^41^

For this kinetic analysis, where we obtain *C* from θ_TIR_, we emphasize the importance of measuring θ_TIR_ in the very same surface region as θ_SPR_ is measured. It would obviously be questionable to draw any conclusion about the influence from liquid exchange when measuring the bulk RI at another location on the chip or in another flow channel. In our system, only a small error is expected by the time delay (<1 s) during the goniometric scan when the instrument moves from the TIR angle region in the angular spectrum to the SPR reflectivity minimum.^17^

**Figure 4:**
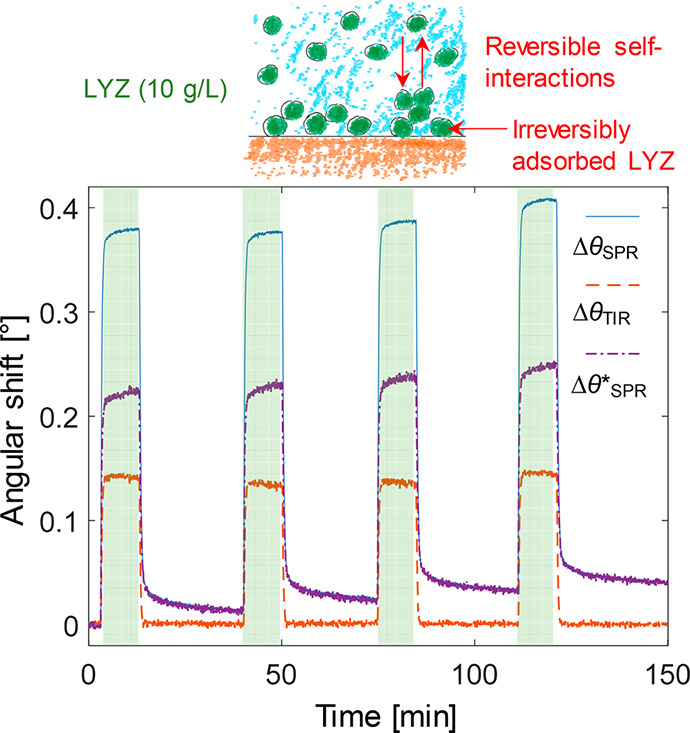
Detecting lysozyme self-interactions. Repeated injections of LYZ (10 g/L) are performed to a gold surface already covered with LYZ (but not PEG). Even after removing the bulk response, a high signal remains, corresponding to more than an additional monolayer of proteins.

As a final result, we show that our bulk response correction reveals self-interactions between LYZ molecules on a plain gold surface. Figure 4 shows the signals in θ_SPR_, θ_TIR_, and θ*_SPR_ when exposing a gold sensor on which a monolayer of LYZ has already been formed. This monolayer was formed simply by exposing the clean gold surface to LYZ, and the coverage of irreversibly adsorbed protein was estimated to 85 ng/cm^2^ (Figure S3). We consider this to be very reasonable for a densely packed monolayer of LYZ on gold as it is a relatively small protein. Remarkably, when injecting more LYZ over this surface and after performing the bulk response correction, a considerable signal remains (Figure 4). Note that in this situation, the “commercial” bulk response correction (*d* → 0) is quite accurate, and it represents the *highest* possible bulk response contribution. (Recall the negative signals due to overcompensation in Figure 2.) Hence, as the signals remain after bulk correction, the only explanation is that LYZ in solution is binding to adsorbed LYZ.

The self-interactions are mostly reversible as the baseline is almost entirely recovered when rinsing. Furthermore, the shift in θ*_SPR_ when injecting 10 g/L is ∼50% higher than the shift corresponding to the LYZ monolayer irreversibly stuck on gold (acting as the receptor layer), showing that multilayers are formed. It is well-known from previous investigations in bulk that lysozyme proteins interact via a weak, short-range attractive potential.^42−44^ Depending on the lysozyme solution conditions, these attractive self-interactions not only lead to a metastable liquid–liquid phase separation,^43,44^ but are also the driving force for the formation of the small equilibrium clusters discussed in relation to Figure 2. Our findings of weakly attractive self-interactions in LYZ are thus not surprising, but they do show that protein self-interactions can be studied by SPR, and once more, the importance of removing the bulk response accurately is highlighted. We find it particularly interesting to consider the possibilities of monitoring the *kinetics* of protein self-interactions by SPR in this manner, which is not possible by static scattering techniques in bulk. Figure 4 also shows that the kinetics of LYZ self-interactions are clearly different compared to the LYZ-PEG interaction, showing for instance a considerably slower dissociation phase.

## Conclusions

We have presented a simple and accurate analytical formula for correcting the bulk response in SPR sensing without the need of a reference channel. The bulk response correction is clearly necessary when working with low affinities because the injected concentrations are high enough to generate unavoidable bulk signals, even if the system has perfect stability with respect to temperature, etc. We have shown that the correction must be performed in a way that takes into account the dimensions of the receptor construct on the surface. We emphasize that simply using the built-in method in commercial instruments will lead to inaccurate results in many cases. Our method assumes an effective decay length for the evanescent field, which may not be accurate for dense organic films, but in that case it can easily be extended to full Fresnel models (see discussion in Supporting Information). The only limitation of our method is that the noise level in the kinetic time trace increases because the corrected SPR response is generated from the TIR angle, which has a higher noise level than the SPR angle. Future work may implement more precise measurements of the RI of the bulk solution,^45^ but it needs to be measured at the sensing spot to avoid artifacts from the liquid exchange process influencing the kinetics. Notably, the metal film thickness may be altered^5^ to improve the resolution in the TIR angle, although this will also influence the resolution in the SPR angle.

We analyzed the interaction between poly(ethylene glycol) brushes and the protein lysozyme at fully physiological conditions, i.e., an ordinary PBS buffer, and found an equilibrium affinity of ∼200 μM for these particular PEG brushes. We also concluded that the system behaves as if it is in constant equilibrium and that the interaction is short-lived. Future work may investigate how the interaction depends on PEG brush properties and environmental factors such as pH or temperature. In addition, we have shown that LYZ interacts reversibly with irreversibly adsorbed copies of itself already at concentrations of 10 g/L. This shows that SPR can be used in a very simple manner to study oligomerization of proteins, which indeed occurs at concentrations which are so high that bulk response correction becomes necessary for accurate results.

## Supporting Information Available

Additional figures, description of how to implement Fresnel models, and a table (PDF)
