# Supplementary material for: Accurate Correction of the “Bulk Response” in Surface Plasmon Resonance Sensing Provides New Insights on Interactions Involving Lysozyme and Poly(ethylene glycol)
Source: ACS Sens. 2022 Mar 17;7(4):1175–82. doi: 10.1021/acssensors.2c00273 (PMC9040059; doi:10.1021/acssensors.2c00273)
Supplement: Supplementary file 1 — se2c00273_si_001.pdf [file se2c00273_si_001.pdf]

# Accurate Correction of the "Bulk Response" in Surface Plasmon Resonance Sensing Provides New Insights on Interactions Involving Lysozyme and Poly(ethylene glycol)

*Justas Svirelis,[1] John Andersson,[1] Anna Stradner[2] and Andreas Dahlin.*[1]*

1 Department of Chemistry and Chemical Engineering, Chalmers University of Technology, 41296 Gothenburg, Sweden.

2 Division of Physical Chemistry, Lund University, SE-22100 Lund, Sweden.

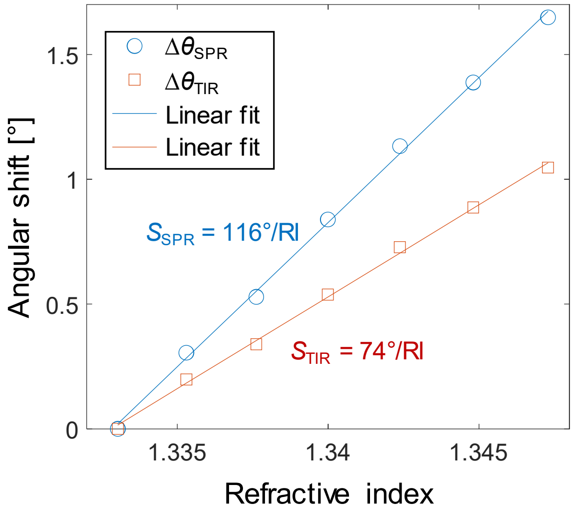


**Figure S1** Refractometric calibration by mixtures of glycerol in water (up to 12% by mass, 2% increments). The linear fits to the data point define the sensitivities of the SPR and TIR angles. Refractive index values were taken from literature.[S1]

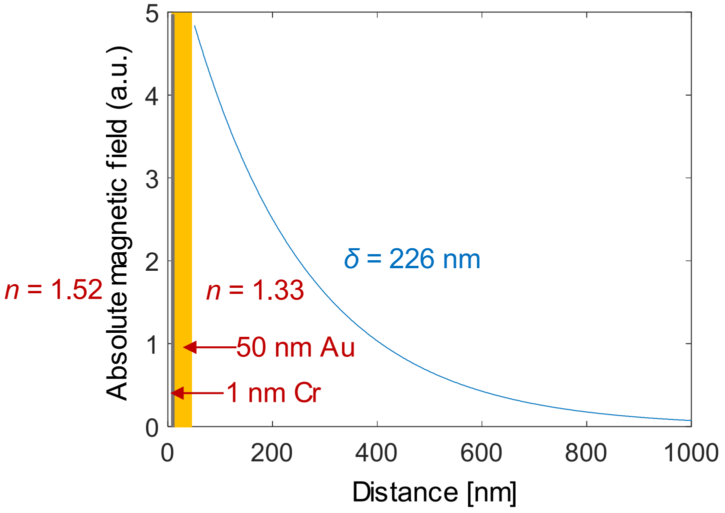


**Figure S2** Simulated field of the surface plasmon at 670 nm. The exponential decay shows a decay length of 226 nm. Details of the calculations are given in previous work.[S2]

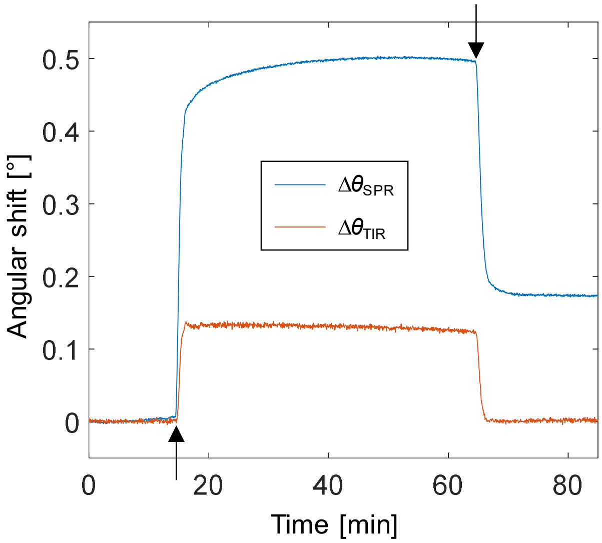


**Figure S3** Control experiment with LYZ (10 g/L) introduced to a clean gold surface. The kinetics of the SPR and TIR angles are now clearly different. The irreversibly bound amount is 0.17°. Since the binding occurs in a thin layer directly on the metal, a simplified formula can be used for quantification.[S3] Using the same values for $S_{SPR}$ and $\delta$ as in the other analysis, this gives a coverage of 85 ng/cm$^2$. The refractivity of the protein is taken from the data in Figure S5. (Arrows indicate injection and rinsing.)

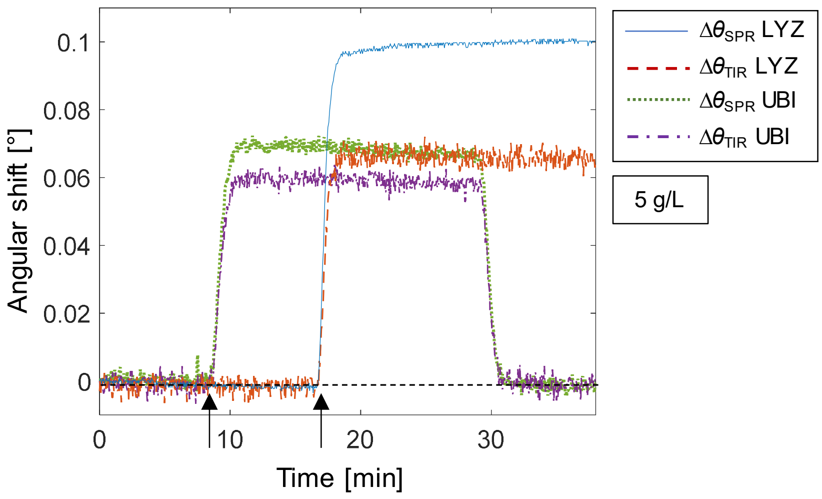


**Figure S4** Control experiment with ubiquitin (UBI). When injected at the same concentration as LYZ (5 g/L) to PEG brushes, the TIR angle responses are similar, but the SPR angle response is higher for LYZ, which again confirms its interaction with PEG. Also, UBI was injected to a PEG brush that had not been exposed to any other protein, but still the baseline was recovered upon rinsing. In contrast, when the PEG brushes were exposed to LYZ for the first time, some proteins remained adsorbed (see main text). (Arrows indicate protein injections.)

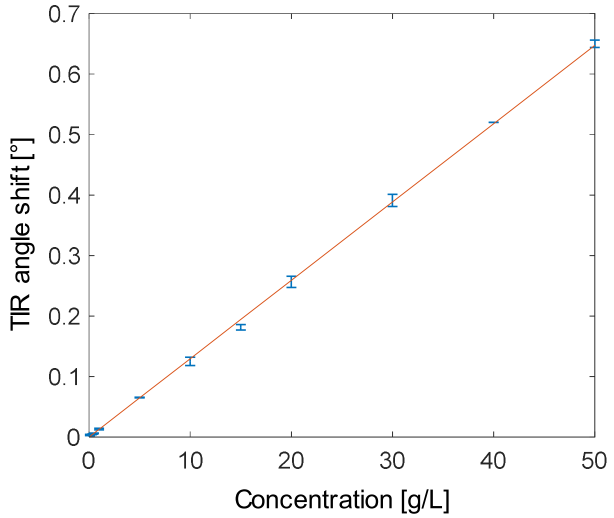


**Figure S5** Plot of TIR angle signals vs injected protein concentration. Error bars are 2 standard deviations. (Error bars that have zero height are due to limited resolution in the TIR angle or few experimental repeats, see Table S1.) The relation is linear with a fitted slope of 0.0128 degrees per concentration increment. Using $S_{\mathrm{TIR}}$ = 74° per RI unit, this translates to a refractometric constant of 0.173 $cm^3/g$ for LYZ in PBS buffer (at 670 nm).

**Extension to full Fresnel models for dense receptor films**

If the existing layer on the surface that the target molecules bind to is very dense (refractive index ~1.4 or more), a single exponential decay function does not describe the evanescent field very well. (In principle, there is a separate decay length in each layer.) In this case the bulk response correction must be performed by using full Fresnel models.[S4] Still, the principle for how to subtract the bulk response is the same and the algorithm is quite similar:

- Determine the parameter $d$, i.e. the excluded zone thickness.
- Use Fresnel fits to initial spectra to get the RI of the layer with thickness $d$.
- Use the TIR angle shift upon injection of molecules to determine $n_0$ by Snell's law.
- Use Fresnel models to calculate the angular spectrum without any surface binding.
- Compare with the experimental spectrum: the extra shift in SPR angle is due to binding.

This approach may also be used if the SPR response is so high that it goes out of the linear regime (see Figure S1). This could be the case if a completely different solvent is injected.

| $C$ [g/L] | $C$ [mM] | Repeats |
|---|---|---|
| 0.001 | 0.000069 | 1 |
| 0.005 | 0.00035 | 1 |
| 0.01 | 0.00069 | 1 |
| 0.02 | 0.0014 | 1 |
| 0.05 | 0.0035 | 1 |
| 0.1 | 0.0069 | 9 |
| 0.2 | 0.014 | 2 |
| 0.3 | 0.021 | 2 |
| 0.4 | 0.028 | 2 |
| 0.5 | 0.035 | 5 |
| 1 | 0.069 | 16 |
| 5 | 0.35 | 2 |
| 10 | 0.69 | 6 |
| 15 | 1.0 | 2 |
| 20 | 1.4 | 4 |
| 30 | 2.1 | 2 |
| 40 | 2.8 | 3 |
| 50 | 3.5 | 2 |

**Table S1** Number of measurements included in the affinity analysis for each LYZ concentration tested, expressed in mass or moles.

**References**

S1 Hoyt, L. F. New table of the refractive index of pure glycerol at 20 degrees C. *Industrial and Engineering Chemistry* **1934**, 26, 329-332.

S2 Dahlin, A. B.; Mapar, M.; Xiong, K. L.; Mazzotta, F.; Hook, F.; Sannomiya, T. Plasmonic nanopores in metal-insulator-metal films. *Advanced Optical Materials* **2014**, 2 (6), 556-564.

S3 Emilsson, G.; Schoch, R. L.; Feuz, L.; Hook, F.; Lim, R. Y. H.; Dahlin, A. B. Strongly stretched protein resistant poly(ethylene glycol) brushes prepared by grafting-to. *ACS Applied Materials & Interfaces* **2015**, 7 (14), 7505-7515.

S4 Ferrand-Drake del Castillo, G.; Emilsson, G.; Dahlin, A. Quantitative analysis of thickness and pH actuation of weak polyelectrolyte brushes. *Journal of Physical Chemistry C* **2018**, 122 (48), 27516-27527.
